# Novel Highly Efficient Buried Gratings for Selective Coupling of SPP Waves onto Single Interfaces

**DOI:** 10.3390/nano14100878

**Published:** 2024-05-18

**Authors:** Arif Nabizada, Hamed Tari, Alessandro Bile, Eugenio Fazio

**Affiliations:** Department of Fundamental and Applied Sciences for Engineering, Sapienza Università di Roma, Via Scarpa 16, 00161 Rome, Italy; arif.nabizada@uniroma1.it (A.N.); hamed.tari@uniroma1.it (H.T.); alessandro.bile@uniroma1.it (A.B.)

**Keywords:** surface plasmon polariton, grating, grating coupling, buried grating, plasmonics, nano-optics, nanophotonics, simulation

## Abstract

Diffraction gratings have always been used to effectively couple optical radiation within integrated waveguides. This is also valid for plasmonic structures that support Surface Plasmon Polariton (SPP) waves. Traditional gratings usually excite SPP waves at the interface where they are located or, for thin metal nanostrips, at both interfaces. But reducing the thickness of the metal layer in the presence of a grating has the handicap of increasing the tunnelling of light towards the substrate, which means higher losses and reduced coupling efficiency. In this paper, we design and optimize novel gratings buried within the metallic thin films for selective coupling of SPP waves onto individual interfaces. Compared with traditional superficial gratings, the novel buried ones demonstrate higher efficiency and much lower residual tunnelling of light through the coupling structures.

## 1. Introduction

Surface Plasmon Polariton (SPP) waves are collective oscillations of electrons and electromagnetic waves (in a TM polarization regime) that propagate along interfaces between metals and dielectric materials [1]. Thus, the interfaces act as waveguides for SPP waves, giving rise to a high transverse confinement as evanescent waves within both the metallic and insulating layers [2]. Plasmonic waves have an important potential for their applications in ultra-compact photonic integrated circuits with high bandwidth and high data-transfer rates [3,4].

Excitation of SPPs can be achieved using traditional light-coupling techniques into waveguides [5,6]. Among them, diffraction gratings would appear to be the most versatile technique, thanks to an efficient coupling and high level of integration. Indeed, gratings can be easily integrated into individual active and passive devices or into complex chips [7,8]. In addition, a grating structure helps to effectively couple SPP waves in dielectric materials with high refractive indices at low wavelengths and small angles.

In recent years, plasmonic circuits are increasingly used together with nonlinear materials to add nonlinear responses and therefore create ultra-compact systems capable of processing and storing information, until the novel frontier of all-optical learning [9,10]. In this context, the use of coupling gratings that can control both coupled and uncoupled light is crucial, in order to avoid unwanted behavior due to residual light that can wander into the substrate or cladding [11].

For these reasons, in this paper we studied a novel grating architecture that can send light selectively onto individual interfaces of thin-film metal waveguides (i.e., of the upper or lower interface, or, if necessary, onto both), strongly reducing the residual transmission of uncoupled light [12]. Particular attention was paid to the coupling at the bottom interface of a metal nanostrip, for applications in solitonic interconnections between two distant SPP waveguides [13,14]. In fact, such new gratings can find important applications in integrated photonic–plasmonic circuits, reaching high power-coupling values in plasmonic waves and strongly limiting the amount of residual, uncoupled light that remains trapped in the circuit and which can give rise to unwanted spurious phenomena.

## 2. Materials and Structure Design

All simulations were performed with COMSOL Multiphysics software (Version 6.2), using the specific physical modules of Electromagnetic Waves in the Frequency Domain. Furthermore, all materials were selected from the internal material library. The simulation procedure involved an integration box surrounded by a Perfectly Matched Layer (PML) to eliminate all numerical reflections. A Gaussian laser beam was introduced through an upper door and directed towards the structure to be analyzed. A fixed mesh was used for the rectangular zones and a free tetrahedral mesh for the thin layers in the lattice zones. The light powers were measured experimentally by introducing specific detectors into the simulator, both for the free and the coupled radiations.

Keeping in mind that one of our goals was to reduce the transmission on uncoupled light, we based the design of the gratings on the following two functional paradigms, apparently contradicting each other:

- the metal layer must be thick enough to eliminate spurious transmission;
- the metal layer must be thin enough to couple the light to the lower interface.

**Figure 1:**
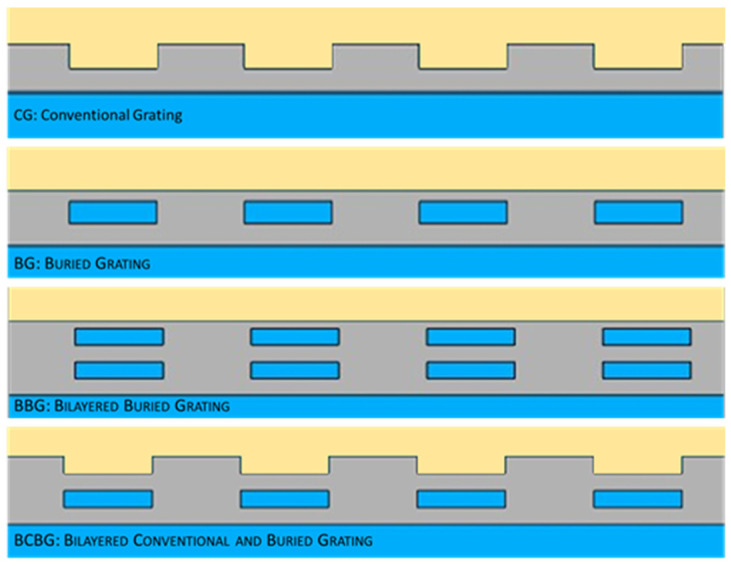
The studied geometries: (CG) Conventional Grating, (BG) Buried Grating, (BBG) Bilayered Buried Grating, and (BCBG) Bilayered Conventional Buried Grating. The substrate (blue) is silicon dioxide, the metal strip (grey) is silver, and the cladding (light orange) is PMMA.

The initial reference of all gratings and performances was still constituted by a traditional one (Figure 1: CG Conventional Grating), realized by corrugating a silver film on an insulating substrate (silicon dioxide). A poly-methyl methacrylate (PMMA) cladding covered the whole structure. 

Starting from conventional gratings and following the design paradigms, we modified the architecture of the grating by initially drowning the corrugation, i.e., by inserting periodic insulating islands within the metal layer (Figure 1: BG Buried Grating). Such configuration provides two metallic layers above and below the grating grooves, which should theoretically reduce the spurious transmission.

In order to calibrate the light resonance through the metal and enhance the coupling at the lower metallic interface, we slightly modified the previous geometry by inserting a double layer of buried islands inside the metal (Figure 1: BBG Bilayered Buried Grating). 

The introduction of a transverse periodicity of the grating within the metal allows a selectively control of the residual transmission, and therefore the coupling of the light to the lower interface of the metal, by means of transverse resonance.

Since, as we will see, the conventional gratings almost always show higher coupling efficiencies than buried gratings, the evolution of the double-layer geometry is now represented by the union of a traditional superficial grating and an underlying buried one in order to enhance its coupling efficiency (Figure 1: BCBG Bilayered Conventional and Buried Grating). In practice, the upper buried islands of the BBG were opened to form traditional superficial grooves, leaving the second buried layer of insulating islands unmodified.

In the simulations, we used the material parameters reported in Table 1.

For SPP waves propagating along the interface between a metal and a dielectric substrate, the longitudinal wave vector obtains the expression [18] reported in Equation (1):

(1) $$\beta_{x}=k_{0}\sqrt{\frac{\varepsilon_{M}^{\prime }\cdot \varepsilon_{SUB}}{\varepsilon_{M}^{\prime }+\varepsilon_{SUB}}}\cdot \left[1+i\frac{\varepsilon_{M}^{\prime \prime }}{2\varepsilon_{M}^{\prime 2}}\cdot \frac{\varepsilon_{M}^{\prime }\cdot \varepsilon_{SUB}}{\varepsilon_{M}^{\prime }+\varepsilon_{SUB}}\right]$$

where $\varepsilon_{M}^{\prime }$ and $\varepsilon_{M}^{\prime \prime }$ are the real and imaginary components of the metal dielectric constant, respectively, while $\varepsilon_{SUB}$ is the real dielectric constant of the substrate, and $k_{0}$ is the wave vector of light in vacuum.

Thus, the wave vector of the input light beam, suitably modified by the grating, must match the SPP wave vector $\beta_{x}$ of Equation (1). 

**Figure 2:**
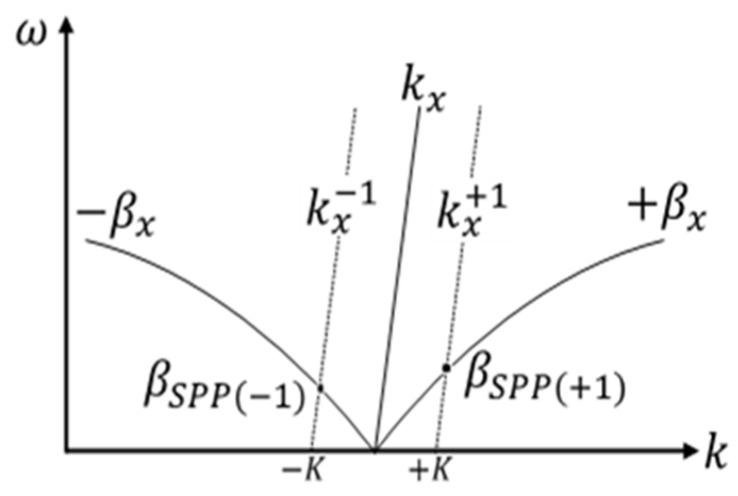
The coupling condition is satisfied where the wave vector of the SPP (±*β_x_*) coincides with the wave vector of the input light modified by the grating in both the forwards $k_{x}^{+}$ and $k_{x}^{-}$ directions [20].

Calling $k_{x}=k_{0}sin\;\theta$, the component of the incident wave vector along the direction of the metallic surface, the grating has the role to transfer its wave vector *K* = 2π/Λ (being Λ the grating wavelength) to the input light according to the following relation [19]:

(2) $$k_{x}^{\pm }=k_{x}\pm mK$$

where m is an integer (*m* = 1,2,3….). Depending on whether *m* is positive or negative, the grating can form SPP waves along both the positive (forward) and negative (backward) *x* directions with the same angle θ. There will be light coupling in the form of an *SPP* wave ($\beta_{SPP\pm 1}$) when

(3) $$k_{x}^{\pm }=\pm \beta_{x}$$

as shown in Figure 2.

Therefore, setting the input angle on the grating means fixing the wave vector of plasmonic propagation: we initially optimized all the structures for 30° of incidence angle (in order to optimize all structures for an angle smaller than the critical one); later on, all possible angles were checked too, as will be shown soon.

## 3. Results and Discussion

**Figure 3:**
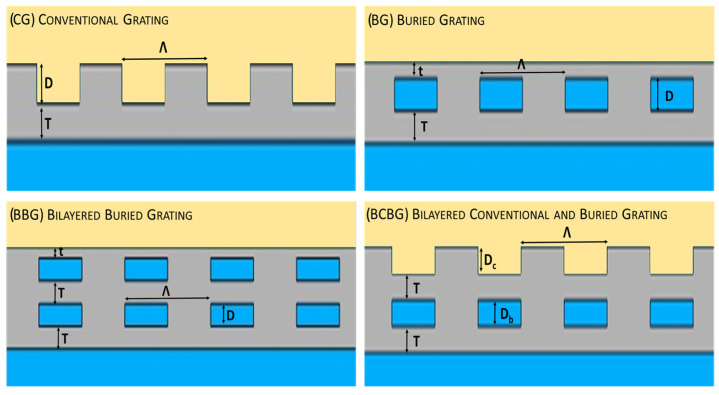
Grating optimization by fine tuning of grating periodicity (Λ); groove depths D, Dc, and Db; and metal layer thicknesses below (T) and above the grooves (t).

The optimization procedure involved an analysis of the material thicknesses and lattice periodicities, as shown in Figure 3.

The objective function F of the optimization process involves maximizing the coupled power on each individual metal interface (i.e., either on the lower one between the metal and the substrate, or on the upper one between the metal and the cladding) together with the minimization of the transmitted power of uncoupled radiation:

(4) $$F=\frac{P_{coupled}}{P_{transmitted}}\;.$$

**Figure 4:**
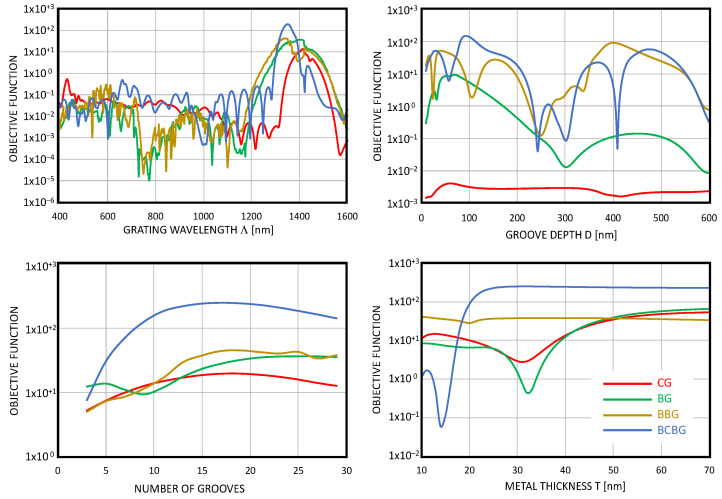
Comparison of the objective functions of the four analyzed structures varying the grating wavelength Λ, the groove depth D, the number of used grooves, and the metallic thickness T below the grooves.

Figure 4 shows the optimization procedure of all the described gratings for the input angle or 30° in the forward propagation. As can be seen, all three buried structures (BG, BBG, and BCBG) had a better performance with respect to the conventional grating CG in red by means of higher values of the objective functions.

Among the gratings, the BCBG grating (blue lines in Figure 4) always demonstrated a much better performance than the others (highest values of the objective function).

The analysis of the behaviors of the gratings shown in Figure 4, and in particular that of BCBG, highlights that the best performance of the grating corresponded to relatively wide peaks or large zones: this leads us to state that the proposed structures show a good tolerance to small variations in thicknesses, the relative responses of which are not so critical.

**Figure 5:**
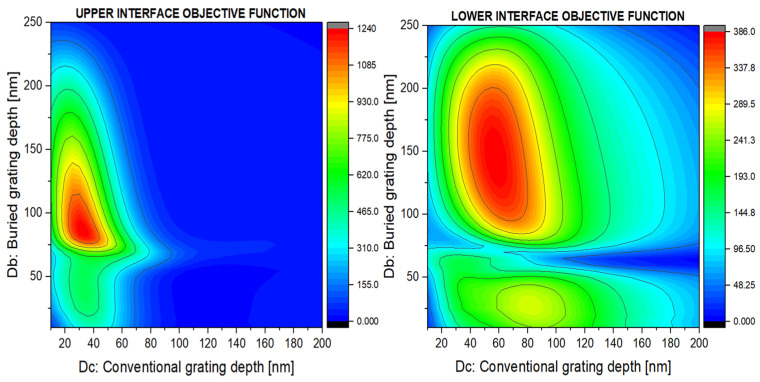
Optimization of the thicknesses of the grooves of the surface grating (Dc) and of the buried grating (Db) for BCBG-type gratings. The two plots refer to the optimization for the processes of coupling light in the form of an SPP wave to the upper (cladding–metal) and lower (metal–substrate) interfaces of the conductive layer.

The BCBG optimization required a fine and selective tuning of the Dc and Db thicknesses, as shown in Figure 5, where the objective function surface plots are reported for the SPP coupling at the upper and lower metal-insulating interfaces.

As can be seen, the objective function reached higher values for the coupling on the upper interface compared to the lower one. However, the working regions are well defined and localized.

**Figure 6:**
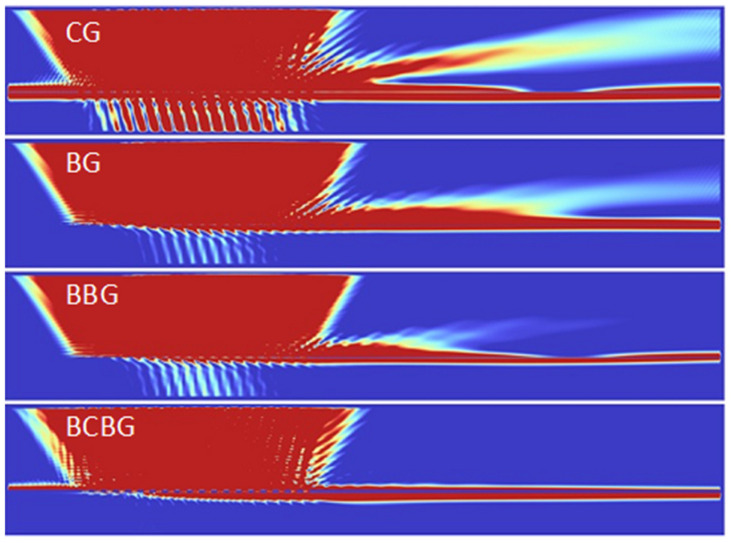
Behaviors of the optimized structures for 30° coupling in the forward direction. CG shows the highest coupling of light as SPP but the highest transmission too. The best performances have been found for the BCBG, which has a slightly lower SPP coupling with respect to CG, but the lowest residual transmission.

Figure 6 shows the performance comparison of the four optimized structures in terms of the SPP coupling and transmitted residual light.

As can be seen, all gratings had a good SPP excitation, but only the last BCBG structure provided a quite efficient suppression of the residual transmission.

Following similar procedures, we optimized all the structures for the input angles of 0°, 30°, and 60° both in forward and backward propagations. The obtained results are reported in Table 2.

Among all cases shown in Table 2, the best performance occurred at 30° for backward propagation, for which the BCBG showed the highest coupling efficiency (30.3), higher than any other coupling obtained for the traditional CG (highest efficiency: 21.1% at 30° backward). However, in this case the residual transmission was relatively high, reaching the value of 5%: indeed, the objective function obtained a relatively low value: F=5.2. We therefore asked ourselves whether it is possible to obtain a drastic decrease in transmitted light even with a small and acceptable reduction in the coupling efficiency.

**Figure 7:**
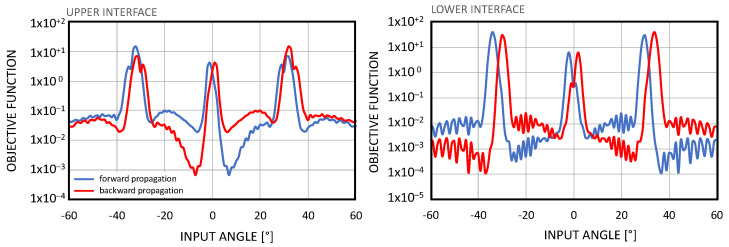
Optimization of the BCBG structure to obtain the highest objective function with the highest possible coupling efficiency. In this case the best performance was found for 34° input, for which the coupling efficiency was 11.4% with a residual transmission as low as 0.3% (that corresponds to the highest objective function F = 40.6 on the left-hand-side). In this case the structure had Λ = 1364 nm, T = 30 nm, D_b_ = 50 nm, and D_c_ = 98 nm.

For this reason, we performed a fine tuning of the BCBG structure, varying the input angle, as shown in Figure 7. The best result for the lower interface was found at 34°, at which the coupling efficiency reduced to 11.4% but the residual transmission dropped down to 0.3% (reaching F = 40.6).

## 4. Conclusions

This study aimed to increase the coupling efficiency and above all to reduce the residual transmission. We studied new grating architectures to couple light into metallic waveguides that support surface plasmon polariton waves. In order to reduce transmission and couple light selectively onto the top or bottom interfaces of a metallic thin film, we investigated the possibility of embedding the grating grooves within the metal as islands of insulating material. Among the three proposed structures, the bilayered conventional buried gratings showed better performance even with respect to the traditional gratings. 

The BCBG gratings demonstrated the highest coupling efficiency with a 30° input, for which 30.3% of the input power was coupled into a back-propagating SPP wave (with a 5% residual light transmitted), versus 21.1% coupled with a traditional grating (with 6.8% of residual transmitted light). 

By slightly varying the input angle from 30 to 34°, the coupling efficiency decreased slightly to a value of 11.4% but the residual light almost disappeared, reaching 0.3%.

The new proposed gratings open up important applications in integrated photonic-plasmonic circuits, reaching high power-coupling values in plasmonic waves and strongly limiting the amount of residual, uncoupled light that remains trapped in the circuit and which can give rise to unwanted spurious phenomena.

## Figures and Tables

**Table 1:** Material parameters used.

Wavelength [nm]	Propagation Region [um]	*ε_PMMA_* [15]	*ε_Ag_* [16]	$\varepsilon_{SiO_{2}}$ [17]
1064	80	2.1928	−47.102 + i 3.2503	2.1524

**Table 2:** Best performance of the four grating structures for different input angles. Here, *k_SPP_* is the coupling efficiency at the lower interface (metal–substrate), *T* is the residual transmission, and F is the objective function in Equation (4). All numbers are percentages.

	0°			30° Forward			30° Backward			60° Forward			60° Backward		
	$\eta_{SPP}$	T	F	$\eta_{SPP}$	*T*	F	$\eta_{SPP}$	*T*	F	$\eta_{SPP}$	*T*	F	$\eta_{SPP}$	*T*	F
CG	16.5	4.8	3.5	9.2	5.8	1.6	21.1	6.8	3.1	0.0	12.1	0.0	21.9	13.4	1.6
BG	6.6	8.6	0.77	1.9	1.0	1.9	13.5	3.0	4.5	0.3	11.5	0.02	28.0	10.1	2.8
BBG	3.7	10.4	0.4	3.2	1.2	2.5	8.9	3.2	2.7	0.2	7.4	0.03	18.1	10.8	1.9
BCBG	13.7	2.1	6.5	9.3	2.2	4.2	30.3	5.8	5.2	0.6	2.5	0.02	27.2	5.7	4.8

## Data Availability

The research data were generated by the direct application of the model and of its associated equations.
